# Flowable Backfill Materials from Bottom Ash for Underground Pipeline

**DOI:** 10.3390/ma7053337

**Published:** 2014-04-25

**Authors:** Kyung-Joong Lee, Seong-Kyum Kim, Kwan-Ho Lee

**Affiliations:** Department of Civil Engineering, Kongju National University, Cheonan 330-717, Korea; E-Mails: joong139@kongju.ac.kr (K.-J.L.); tjdrua0614@kongju.ac.kr (S.-K.K.)

**Keywords:** backfill materials, bottom ash, small-scaled chamber test, controlled low strength materials

## Abstract

The purpose of this study was to investigate the relationship between strength and strain in manufacturing controlled low strength materials to recycle incineration bottom ash. Laboratory tests for controlled low strength materials with bottom ash and recycled *in-situ* soil have been carried out. The optimum mixing ratios were 25%–45% of *in-situ* soil, 30% of bottom ash, 10%–20% of fly ash, 0%–3% of crumb rubber, 3% of cement, and 22% of water. Each mixture satisfied the standard specifications: a minimum 20 cm of flowability and 127 kPa of unconfined compressive strength. The average secant modulus (*E*_50_) was (0.07–0.08) *q*_u_. The ranges of the internal friction angle and cohesion for mixtures were 36.5°–46.6° and 49.1–180 kPa, respectively. The pH of all of the mixtures was over 12, which is strongly alkaline. Small-scale chamber tests for controlled low strength materials with bottom ash and recycled *in-situ* soil have been carried out. Vertical deflection of 0.88–2.41 mm and horizontal deflection of 0.83–3.72 mm were measured during backfilling. The vertical and horizontal deflections of controlled low strength materials were smaller than that of sand backfill.

## 1. Introduction

Power consumption is constantly increasing due to the expansion and industrialization of modern cities. In Korea in 2012, 10 domestic thermal power plants produced electricity from thermal generators. Construction of additional thermal power plants is also anticipated. It is expected that over 800 million tons of fly ash, a byproduct of thermal power plants, will be generated in 2015.

Bottom ash is a byproduct of the combustion of pulverized coal in power plants. Bottom ash is a coarse granular material, in contrast to the very fine structure of fly ash. In general, approximately 8%–9% of all generated coal ash is bottom ash. Bottom ash has been reused as a replacement for various construction materials such as cement binder, aggregate, natural sand, and road construction material because of its particle-size distribution characteristics [1]. Bottom ash can be recycled and used efficiently, but appropriate technology is needed to do so.

Controlled low strength material (CLSM) is a mixture of coal fly ash, water, Portland cement, and other construction or recycling materials. CLSM flows like a liquid, sets up like a solid, is self-leveling, and requires no compaction or vibration to achieve maximum density. This extremely versatile construction material has been used in a wide variety of applications. Some of the many successful applications of controlled low strength materials include pavement bases, backfill for retaining walls, culverts, and underground pipe trenches [2]. Flowable fill offers a number of advantages over conventional earth-fill materials, which require controlled compaction in layers. The advantages of flowable fill include ease of mixing and placement and ability to flow into hard-to-reach places, as well as the fill’s self-leveling characteristics [3].

This study investigated the relationship between strength and strain in manufacturing controlled low strength materials to recycle bottom ash generated in power plants into a construction material. To do this, laboratory tests and small-scaled chamber tests were carried out to measure strength and strain according to the curing time and mixing ratio of bottom ash, *in-situ* recycled soil, and crumb rubber and to show the optimum conditions manifesting strength and strain based on the results of measurement. On the basis of test results, this study tries to determine whether incineration bottom ash may be used as a construction material.

## 2. Literature Review

The American Concrete Institute defines a controlled low strength material as a cemented material that is in a flowable state at the time of placement and has a specified compressive strength of 8275 kPa or less at the age of 28 days [4]. The lower-end strengths of 150–700 kPa are used in applications that require excavation for maintenance. A controlled low strength material in the 2000–8000 kPa range is a permanent, lean concrete fill that has a bearing capacity much greater than that of compacted granular soils but less than that of rock. CLSM is mixed and delivered like ready-mixed concrete, except the proportions consist of less Portland cement and more fly ash or aggregate. The resulting mix is more fluid than conventional concrete. The fluid mix hardens into either a stiff soil-like mass or a rock-like mass depending on the mix design [5–9].

The pipe material and shape, and the support of the material beneath and to the sides of the pipe, all affect the maximum load that pipes are capable of carrying [10]. The bedding under the pipe supports vertical loads, the side-fill prevents pipes from deflecting outward, and the haunch zone is a part of both sections. Good support in the haunch zone is very important to carry vertical loads and to prevent lateral deformations. The difficulty of filling and compacting conventional backfill materials in the haunch zone causes large variability in support in this area. However, controlled low strength materials can easily flow into this zone and provide uniform and continuous support to the pipe. In general, if the bedding and backfill are shaped to the contour of the pipe, better support and higher permissible loads are obtained [11–17].

The available load bearing capacity of rigid pipes is typically determined using a three-edge bearing test load. However, the three-edge bearing test represents a severe loading condition. In general, buried pipes are capable of supporting greater loads than those determined by the three-edge bearing test based on their bedding quality and backfill. Spangler determined the load factor (Lf) of pipe soil beddings and presented a corresponding classification system (known as the Marston-Spangler bedding classification) [18]. A load factor is the ratio of permissible field load to three-edge bearing test load. A load factor, greater than unity, indicates that the magnitude of the allowable field loading is greater than that of the test load [10]. The Marston-Spangler classifications are as follows [19]:

- Class D (Impermissible bedding): Little or no effort is taken to shape the bedding to fit the invert of the pipe or to fill the haunch zone. Backfill is partially compacted.
- Class C (Ordinary bedding): Earth bedding is pre-shaped to fit the invert of the pipe for a width of at least 50% of the pipe diameter. The pipe is surrounded by granular materials to a height of at least 0.15 m (0.5 ft) above its crown. The granular materials are shovel placed and shovel tamped to completely fill all spaces under and adjacent to the pipe.
- Class B (First class bedding): The pipe is placed on bedding made out of fine granular materials. The bedding is shaped to fit the invert of the pipe with a template for a width of at least 60% of the pipe diameter. The pipe is surrounded by granular materials to a height of at least 0.3 m (1 ft) above its crown. The granular materials are carefully placed to completely fill the haunch zone and the side-fill area. The granular materials are thoroughly compacted on each side and under the pipe in thin layers not exceeding 0.15 m (0.5 ft) in thickness.

In addition to the Marston-Spangler classifications, Standard Installation Direct Design (SIDD) models were recently adopted by ASCE and AASHTO. The Standard Installation Direct Design differentiates between four types of backfill designs. Type I is a carefully hunched and densely compacted backfill. Type II is a slightly lower quality installation that is approximately equivalent to Class B Marston-Spangler bedding. Type III is roughly equivalent to Class C, and Type IV is roughly equivalent to Class D Marston-Spangler bedding [11].

## 3. Testing Materials and Basic Testing

### 3.1. Materials

The bottom ash used in this research was taken from a power plant in Seocheon, Korea, and added to backfill materials. The gravel-size particles were screened through a standard No. 4 sieve. The effective size (D_10_), uniformity coefficient, and gradation coefficient of the bottom ash were calculated at 0.94, 1.82 and 3.2 mm, respectively. The particle-size distribution of the bottom ash appeared to have the characteristics of poorly graded sand. The bottom ash had a specific gravity of 2.56, and its chemical composition by XRF was 49.8% SiO_2_, 18.2% Al_2_O_3_, 10.4% Fe_2_O_3_, and 13.9% CaO. In general, ground bottom ash can be used as a good pozzolanic material. The increase in shear strength due to the addition of bottom ash to soil mixtures is caused not only by the development of friction at the interface of the mixture components but also by the bond strength due to the pozzolanic reaction of the bottom ash.

In this study, recycled *in-situ* soil from a construction site near Cheonan City, Korea, and fly ash were used. Selected properties of the soils and fly ash are shown in Table 1. The characteristics of the *in-situ* soil were relatively uniform, and its water content was approximately 14%.

The fly ash used in this research was generated at the Tae-An Thermoelectric Power Plant in the Taean Peninsula, where anthracite coal was used for fuel. Until 1997, all fly ash not used as cement admixture had been disposed of in waste ponds at the plant. The fly ash was at the stage of the process just prior to refinement for use in cement mixing. The fly ash was classified as class-F according to the ASTM classification.

Type I Portland cement (ASTM-150) was supplied by Sungshin Industries, Korea, and was used for solidifying the materials. Selected chemical properties of the cement and fly ash are presented in Table 2.

In addition, powder-type crumb rubber was adopted. Its specific gravity is 0.93, and its average particle size is approximately 0.5–1.0 mm. Its tensile strength is 200 kg/cm^2^.

### 3.2. Determination of Optimum Mixing Ratio

To find the point of minimum water demand (PMWD), the quantity of the sand or the soil was fixed in the first stage, while the quantities of cement, fly ash, recycled *in-situ* soil, and water were varied. A flow test and unconfined compressive strength test were then carried out to determine the optimum mixing ratio. A 7.6 cm diameter by 15.2 cm height open-ended cylinder and a 50 cm by 50 cm smooth glass plate were used for the flow test. The open-ended cylinder was placed on the glass plate to simulate a smooth, leveled surface. Given the setting, the cylinder was completely filled with the flowable fill mix, and its surface was leveled off with a straight edge. The cylinder was then quickly lifted and the diameter of the circular section of flowable fill that formed on the glass plate was taken as the spread. The specified flowability was 20–30 cm [20]. Unconfined compressive strength tests were also carried out. From the results of the flow test and unconfined compressive strength tests, the relationship between the water and cement ratio and unconfined compressive strength was obtained. The target strength for the three-day curing specimen is 127 kPa for manual excavation. The optimum mixing ratio should satisfy the specified flowability (20–30 cm) and unconfined strength minimum (127 kPa). While designing a mix, it is also important to consider the ease of handling the flowable fill, the homogeneity of the mix, and the possibility of segregation. The optimum mixing compositions by weight for four different mixtures are shown in Table 3.

### 3.3. Unconfined Compression Test

The unconfined compression apparatus was used in this test. The objective of the testing program was to obtain the undrained elasticity modulus *versus* curing time. The diameter of the test material was 5 cm, and its length was 12.5 cm. The compression velocity was 2 mm/min, which was equivalent to 1.6% strain per minute for the 5 cm diameter samples tested in this study. Cured specimens for 0, 3, 7, 14, and 28 days were used.

Table 4 presents the variation of the unconfined compressive strength of CLSM with time, including the type of soil and curing method (dry and wet). The target values of specification in Korea are over 127 kPa at three days’ curing time and around 980 kPa at 28 days’ curing time. All of the test cases satisfy the specifications.

**Figure 1.**
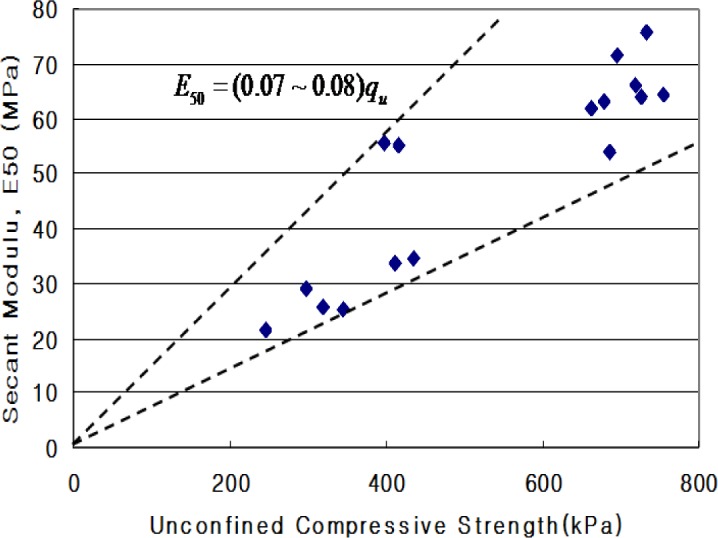
Unconfined compressive strength (*q*_u_) and secant modulus (*E*_50_) of CLSM.

Figure 1 shows the relationship between the unconfined compressive strength (*q*_u_) and secant modulus (*E*_50_) of CLSM. The secant modulus denotes the slope of the line between the origin and the point *q*_u_/2 on the stress-strain curve. Test results indicated that the CLSM had (*E*_50_) values of (0.07–0.08) *q*_u_.

### 3.4. Triaxial Test

**Figure 2.**
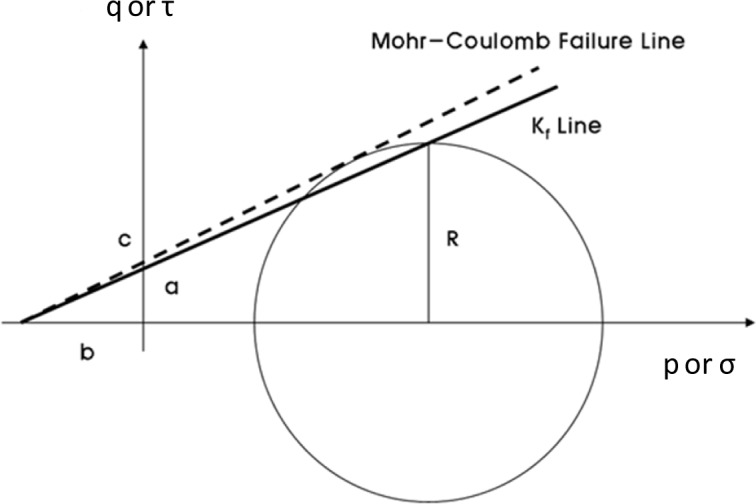
Relationship between the K_f_ line and Mohr-Coulomb plot.

**Figure 3.**
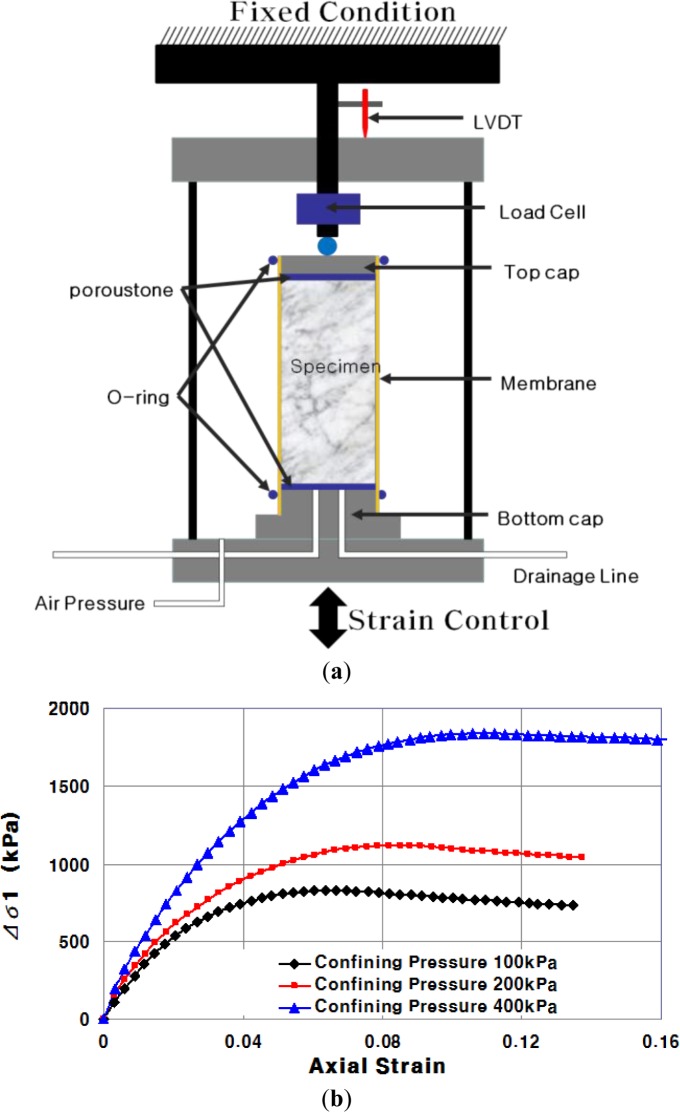
(**a**) Triaxial testing equipment and (**b**) typical test results.

The triaxial test was conducted to obtain the cohesion and internal friction angle of each mixture. At failure, the Mohr’s circle will touch a line that is the Mohr-Coulomb failure envelope; this makes an angle with the normal stress axis. A situation in which the points with p’ and q’ coordinates of all the Mohr’s circles are joined is called a stress path (K_f_). The relationship between the stress path line and the Mohr-Coulomb plot is shown in Figure 2. The test procedure of UU (unconsolidated-undrained) followed ASTM D 2850-95 [21]. The testing equipment and test results are shown in Figure 3. The test results are shown in Table 5. The measured internal friction angles range from 36.5° to 46.6°, and cohesions of CLSM are 49.1–143.8 kPa.

### 3.5. pH Test

The pH measured for each test specimen, according to KS F 2103, is shown in Table 6. The pH was measured just after mixing and at 28 days’ curing time. The measured values range from 12.26 to 12.62 for just after mixing and from 11.37 to 12.19 for 28 days’ curing time, due to the chemical reaction between calcium hydroxide present in the mixtures and carbon dioxide from the atmosphere [22,23]. The measured values are strongly alkaline due to the cement, fly ash, and bottom ash included in the test specimens.

## 4. Laboratory Chamber Test

### 4.1. Testing Instruments and Setup

**Figure 4.**
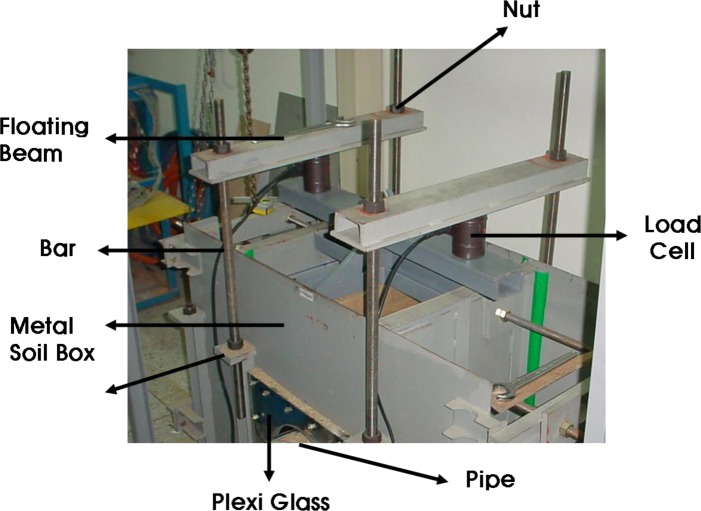
Small-scale chamber.

**Figure 5.**
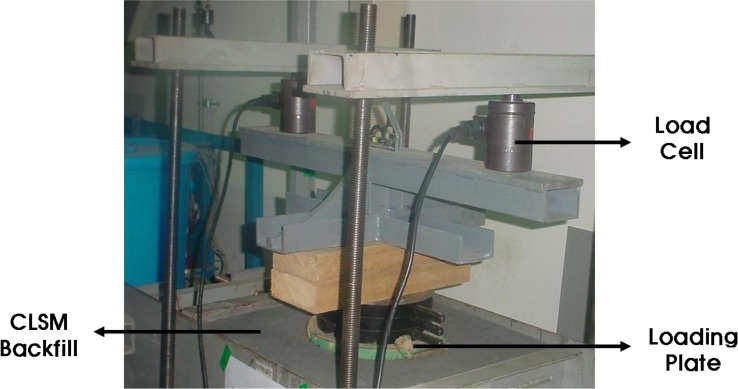
Loading system.

**Figure 6.**
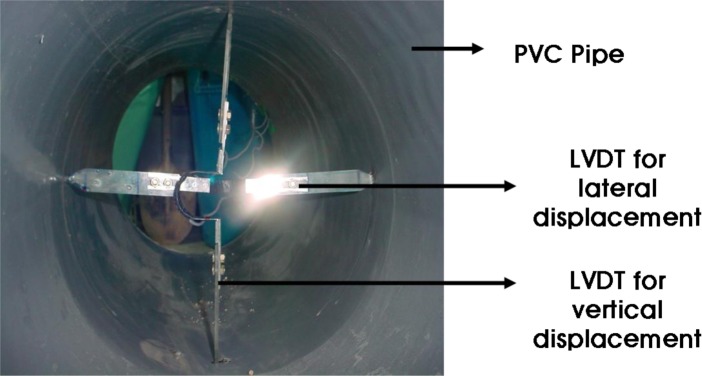
LVDT.

**Figure 7.**
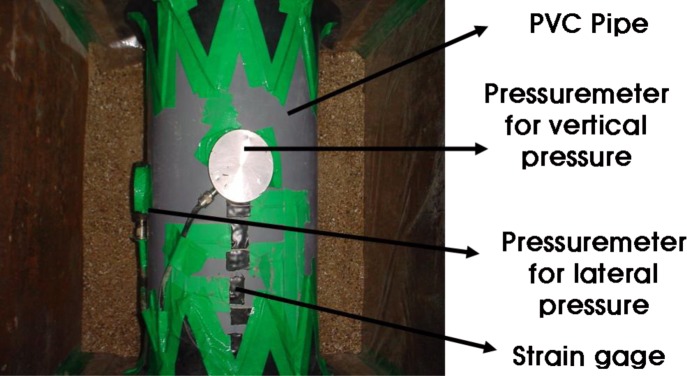
Pressuremeter.

**Figure 8.**
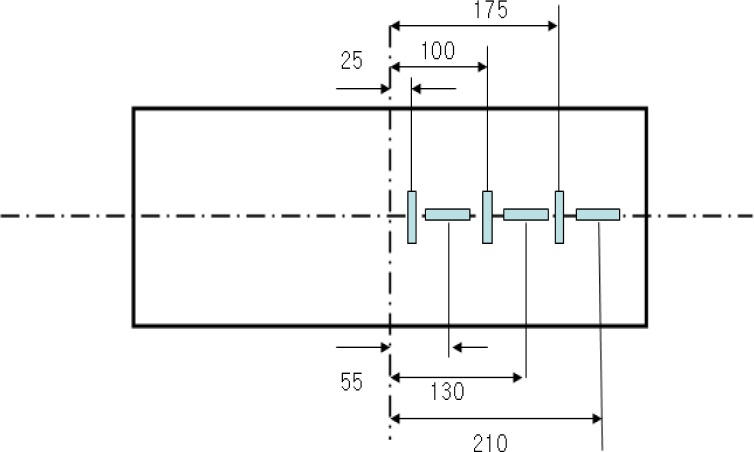
Strain gauge on PVC pipe.

The A small-scale chamber test was carried out to evaluate the performance of an underground pipe-CLSM system. The dimensions of the small-scale chamber are 1.4 m × 0.6 m × 0.9 m, and it is reinforced with horizontal and vertical flat metal strips as shown in Figure 4. As observed in the figures, a square metal plate with an elliptical hole is attached to the box with nuts and bolts. A rubber membrane sheet is placed between the chambers and the plate before tightening the plate, and a 20-cm circular hole is cut in the plate to insert the pipe. The membrane is used to ensure water tightness without affecting the behavior of the pipe. The loading system with a floating plate on top of the chamber and two metallic blocks over the plate is shown in Figure 5. A metal beam with a load cell is placed between the metal blocks and the beam. The maximum capacity of each load cell was 2 tons. The load cell was pre-calibrated using a 5-ton universal testing machine. A linear variable differential transformer (LVDT), shown in Figure 6, was installed to obtain the vertical and horizontal deflections of the pipe. Two pressure measurement devices for soil or CLSM, shown in Figure 7, were installed against the pipe at the crown and spring-line. Devices to measure strains in the pipe wall at six equally spaced locations at the center of the pipe were also installed, as shown in Figure 8. An automated data acquisition system was employed for collecting the data. The different conditions of the test are presented in Table 7. Natural sand was used as a bedding material. A polyvinyl chloride (PVC) pipe with a 30 cm diameter and 0.7 cm thickness was installed. Its modulus of elasticity and Poisson’s ratio were 3490 MPa and 0.31, respectively.

### 4.2. Testing Results and Analysis

**Figure 9.**
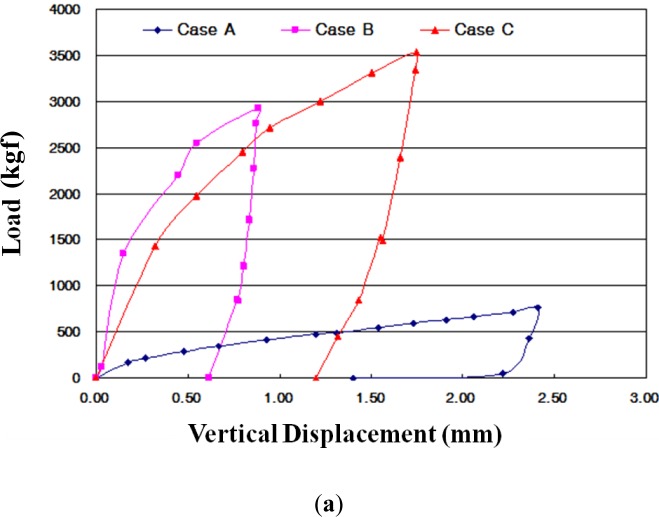

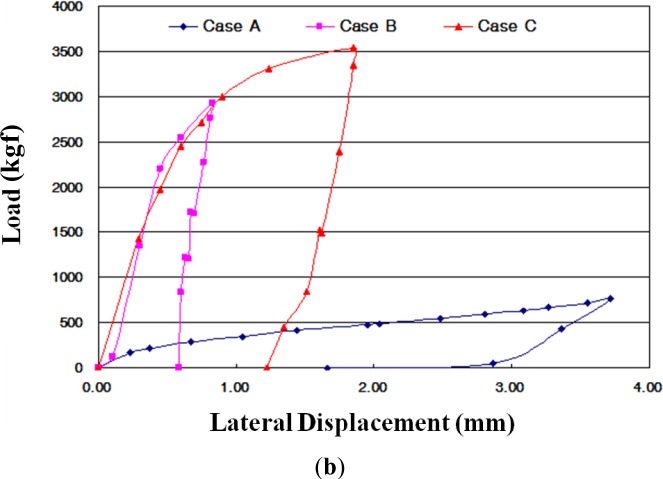
Vertical (**a**) and lateral (**b**) deformation of each case.

The vertical deformations for the three cases are shown in Figure 9. For recycled *in-situ* soil, Case A applied approximately 760 kgf of loading. The applied loading for Case B and Case C was up to 3600 kgf. The measured vertical displacements were 2.41 mm for Case A, 0.87 mm for Case B, and 1.75 mm for Case C. The measured lateral displacements were 3.72 mm for Case A, 0.83 mm for Case B, and 1.85 mm for Case C. The applied load of Case A was just 1/5–1/6 of that of Cases B and C due to large displacement during loading. During the unloading for each case, the rebounded vertical displacements were 1.01 mm for Case A, 0.26 mm for Case B, and 0.55 mm for Case C. The use of crumb rubber in CLSM showed a small effect, giving an elastic property to the mixtures. In particular, the lateral displacement of Case A showed larger than average lateral displacement. This result means that the use of *in-situ* soil had a lower lateral confining effect and that the use of CLSM with bottom ash as backfill material increases the stiffness of flexible pipes.

**Figure 10.**
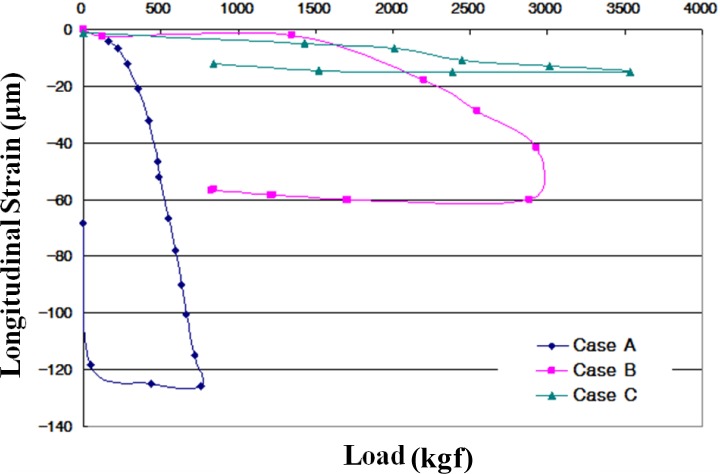
The longitudinal strain of the PVC pipe.

The longitudinal strain of the PVC pipe, shown in Figure 10, was measured as the load was applied. The maximum longitudinal strains were −125.7 μm for Case A; −60.0 μm for Case B and −14.97 μm for Case C. This result means that the use of CLSM with bottom ash decreases the longitudinal strain of the PVC pipe.

**Figure 11.**
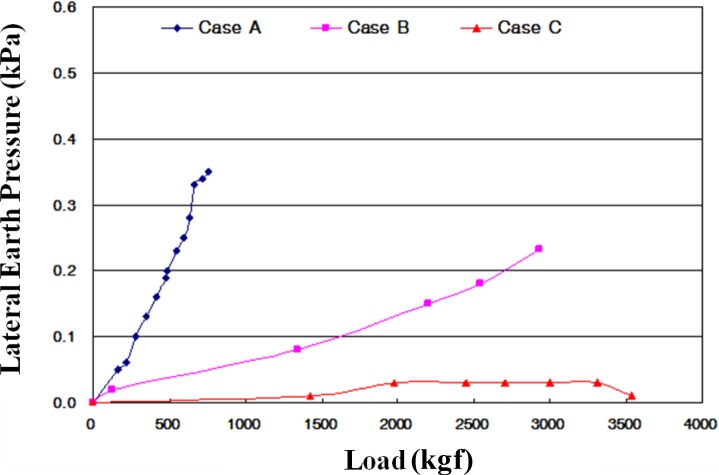
Lateral earth pressure (kPa).

The measured lateral earth pressure for each case is shown in Figure 11. The maximum lateral earth pressures are 0.35 kPa for Case A; 0.23 kPa for Case B and 0.03 kPa for Case C. In the case of flexible pipes such as PVC, the lateral earth pressure depends on the type of backfill material, degree of compaction, and an arching effect. The use of CLSM with bottom ash as backfill material induced a large reduction of lateral earth pressure applied on the underground pipe.

**Figure 12.**
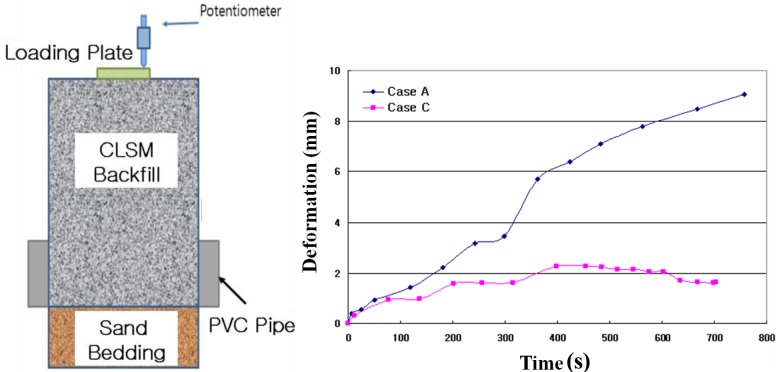
Deformation (mm) at Surface of CLSM backfill

**Figure 13.**
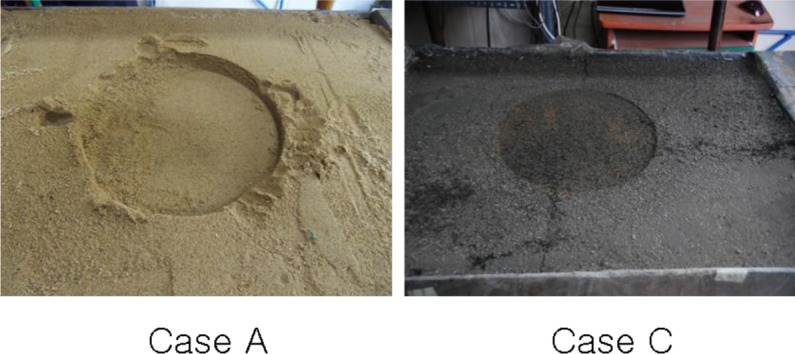
Settlement shape of the surface.

During the loading on the pipe, the deformation of the surface level was measured with loading time (seconds). The testing setup and results are shown in Figure 12. The maximum surface deformations are 9.06 mm for Case A and 2.27 mm for Case C. Case A showed steadily increasing deformation with loading time for *in-situ* soil backfill. On the other hand, Case C showed a peak value of deformation and then decreased deformation with loading time for CLSM with bottom ash and crumb rubber. This finding is due to the hardening effect of cement in CLSM, which is shown in Figure 13.

## 5. Conclusions

The research presented in this paper aimed to characterize the engineering properties of natural soil, bottom ash, and crumb rubber as controlled low strength materials to be used around underground pipe lines. A number of laboratory tests, including triaxial and small-scaled laboratory chamber tests, were carried out to evaluate the index properties and unconfined compressive strength. Despite the possible limitations of laboratory tests, the following conclusions can be drawn:

(1) Based on the flow test and unconfined compressive strength, the optimum mixing ratio was determined. For Case 4 with crumb rubber, the mix ratio of soil, bottom ash, fly ash, crumb rubber, cement, and water by total weight was 32%, 30%, 10%, 3%, 3% and 22%, respectively.
(2) The target values of specifications for unconfined compressive strength in Korea are over 127 kPa at three days’ curing time and within 980 kPa at 28 days’ curing time. All of the test cases satisfy the specifications. The relationship between unconfined compressive strength (*q*_u_) and secant modulus (*E*_50_) in CLSM was analyzed. The secant modulus denotes the slope of the line between the origin and the point *q*_u_/2 on the stress-strain curve. Test results indicated that the CLSM had (*E*_50_) values of 0.07–0.08 *q*_u_.
(3) The UU (unconsolidated-undrained) test procedure followed ASTM D 2850-87. The measured internal friction angles range from 36.5° to 46.6°, and cohesions of CLSM are 49.1–143.8 kPa.
(4) The applied loading for Case B and Case C was up to 3600 kgf. The measured vertical displacements are 2.41 mm for Case A, 0.87 mm for Case B, and 1.75 mm for Case C. The measured lateral displacements are 3.72 mm for Case A, 0.83 mm for Case B, and 1.85 mm for Case C. The applied load of Case A was just 1/5–1/6 of that of Cases B and C due to large displacement during loading.
(5) The maximum lateral earth pressures are 0.35 kPa for Case A, 0.23 kPa for Case B, and 0.03 kPa for Case C. In the case of flexible pipes such as PVC, the lateral earth pressure depends on the type of backfill material, degree of compaction, and an arching effect. The use of CLSM with bottom ash as backfill material resulted in a large reduction of lateral earth pressure applied on the underground pipe.
(6) The maximum surface deformations are 9.06 mm for Case A and 2.27 mm for Case C. Case A showed steadily increasing deformation with loading time for *in-situ* soil backfill. On the other hand, Case C showed a peak value of deformation and then decreased deformation with loading time for CLSM with bottom ash and crumb rubber.
(7) Judging from the extensive testing in the lab, the use of *in-situ* soil, bottom ash, and crumb rubber as CLSM as construction materials in civil engineering can be a potential option to reinforce or stabilize underground pipeline systems.

## Figures and Tables

**Table 1.** Selected properties of the soils and fly ash.

Test	Bottom Ash	*In-situ* Soil	Fly Ash
Specific Gravity	2.56	2.70	2.17
Water Content (%)	3.55	14.06	–
Classification (UIUC)	SP	SP	–
C_u_ (uniformity coefficient)	3.19	5.68	16.44
C_c_ (coefficient of gradation)	1.17	1.18	0.63
D_10_ Size	0.94	0.22	–
D_30_ Size	1.82	0.57	–
D_60_ Size	3.00	1.25	–

**Table 2.** Selected chemical properties of cement and fly ash (Unit: %).

Type	SiO_2_	Al_2_O_3_	Fe_2_O_3_	TiO_2_	SO_3_	CaO	MgO	K_2_O	Na_2_O	P_2_O_5_	Loss of Ignition
Cement	21.80	4.40	2.90	–	2.60	63.2	3.60	–	0.62	–	0.67
Fly Ash	60.33	24.78	3.82	1.06	0.88	2.39	0.84	0.86	0.59	0.50	4.84

**Table 3.** Optimum mixing composition (wt%).

Type	Recycled *in-situ* Soil	Bottom Ash	Fly Ash	Crumb Rubber	Cement	Water
Case 1	45	20	10	–	3	22
Case 2	35	30	10	–	3	22
Case 3	25	30	20	–	3	22
Case 4	32	30	10	3	3	22

**Table 4.** Unconfined compressive strength (kPa).

Type		Curing Time (day)			
		3	7	14	28
Dry Curing	Case 1	319.8	436.1	726.0	754.8
	Case 2	279.3	412.8	720.0	734.0
	Case 3	345.6	416.1	679.5	685.3
	Case 4	247.3	398.4	697.0	663.6

Wet Curing	Case 1	309.1	480.9	616.1	670.9
	Case 2	260.8	413.8	413.1	488.0
	Case 3	395.0	624.1	748.2	838.3
	Case 4	295.1	518.0	535.4	652.0

**Table 5.** Test Results of Triaxial Test.

Curing Day and Method		Parameter			
		Internal Friction Angle	Cohesion (kPa)	a	b
14 day; Dry Condition	Case 1	434	100.8	0.69	73.2
	Case 2	46.6	67.7	0.73	46.5
	Case 3	43.9	93.1	0.69	66.9
	Case 4	40.6	160.2	0.65	121.5

14 day; Wet Condition	Case 1	44.5	121.2	0.70	86.4
	Case 2	44.1	89.0	0.70	63.9
	Case 3	43.1	143.8	0.68	104.9
	Case 4	40.2	150.7	0.65	115.0

28 day; Dry Condition	Case 1	40.6	95.1	0.65	72.2
	Case 2	42.4	49.1	0.68	36.2
	Case 3	41.7	82.9	0.67	61.9
	Case 4	40.5	113.0	0.65	85.8

28 day; Wet Condition	Case 1	43.8	132.0	0.69	95.2
	Case 2	43.5	108.4	0.69	78.5
	Case 3	47.4	97.7	0.74	66.1
	Case 4	36.5	180.7	0.60	145.2

**Table 6.** Test results for pH.

Mixtures	Curing Time (day)	
	Just after Mixing	28 days
Case 1	12.26	11.37
Case 2	12.31	11.67
Case 3	12.62	12.19
Case 4	12.59	11.97

**Table 7.** The mixing composition (wt%).

Type	Recycled Soil	Bottom Ash	Fly Ash	Crumb Rubber	Cement	Water
Case A	100	–	–	–	–	–
Case B	35	30	10	–	3	22
Case C	32	30	10	3	3	22
